# Polymerase Spiral Reaction Assay for Rapid and Real Time Detection of West Nile Virus From Clinical Samples

**DOI:** 10.3389/fcimb.2020.00426

**Published:** 2020-08-27

**Authors:** Priyanka Singh Tomar, Jyoti S. Kumar, Sapan Patel, Shashi Sharma

**Affiliations:** ^1^Division of Virology, Defence Research and Development Establishment, Gwalior, India; ^2^School of Studies in Botany, Jiwaji University, Gwalior, India

**Keywords:** WNV, PSR, env, rapid diagnosis, Flaviviruses

## Abstract

West Nile virus (WNV) is a mosquito**-**borne virus of public health importance. Currently, there is no FDA approved vaccine available against WNV infection in humans. Therefore, the early diagnosis of the WNV infection is important for epidemiologic control and timely clinical management in areas where multiple Flaviviruses are endemic. The present study aimed to develop reverse transcription polymerase spiral reaction (RT-PSR) assay that rapidly and accurately detects the envelope (env) gene of WNV. RT-PSR assay was optimized at 63°C for 60 min using real**-**time turbidimeter or visual detection by the addition of SYBR Green I dye. The standard curve for RT-PSR assay was generated using the 10-fold serial dilutions of *in vitro* transcribed WNV RNA. To determine the detection limit of RT-PSR assay, an amplified product of conventional RT-PCR was *in vitro* transcribed as per standard protocol. The detection limit of the newly developed RT-PSR assay was compared with that of conventional RT-PCR and CDC reported TaqMan real-time RT-PCR using a serial 10-fold dilution of IVT WNV RNA. The detection limit of RT-PSR was found to be 1 RNA copy, which is 100-fold higher than that of conventional RT-PCR (100 copies). This suggests that RT-PSR assay is a valuable diagnostic tool for rapid and real-time detection of WNV in acute-phase serum samples. The assay was validated with a panel of 107 WNV suspected human clinical samples with signs of acute posterior uveitis and onset of febrile illness. Out of 107 samples, 30 were found positive by RT-PSR assay. The specificities of the selected primer sets were established by the absence of cross-reactivity with other closely related members viruses of the Flaviviruses, Alphaviruses, and Morbilliviruses groups. No cross**-**reactivity was observed with other viruses. To best of our knowledge, this is the first report describing the RT-PSR assay for the detection of RNA virus (WNV) in clinical samples. RT-PSR is a high throughput method and more than 30 reactions can be run at once in real-time turbidimeter. PSR assay has potential to be used for a rapid screening of large number of clinical samples in endemic areas during an outbreak.

## Introduction

West Nile virus (WNV) causes numerous outbreaks worldwide and major cases were reported in New York, in 1999 (Lanciotti et al., 1999). It was first isolated from the West Nile region of Uganda in 1937 and has become an important cause of humans and animal disease worldwide (Anderson et al., 1999; Chancey et al., 2015). During outbreaks of emerging infectious viruses, accurate and rapid diagnosis is important for reducing further spread through timely implementation of appropriate antiviral treatments, vaccines and controlling measures (Petersen and Roehrig, 2001; Mukhopadhyay et al., 2003; Solomon et al., 2003; Dauphin and Zientara, 2007; Michaelis et al., 2009; Lim et al., 2011; Zengguo et al., 2016). Outbreaks of WN viral disease in human beings have been reported in Africa, the Middle East, Europe, West and South Asia, Australia, and North America (Lanciotti et al., 1999; Vazquez et al., 2010; Caren et al., 2015; European Centre for Disease Prevention Control (ECDC)., 2017). WNV can now be found in many avian and mosquito species throughout North America. From 1999 to 2010, more than 2.5 million people were infected with over 12,000 reported cases of encephalitis or meningitis and over 1,300 deaths (Komar, 2000; Marfin and Gubler, 2001; Kilpatrick, 2011; Jolanta et al., 2018). The outbreak of WNV has been recently reported from south Indian states including TamilNadu and Kerala in 2011 (Anukumar et al., 2011; Kumar et al., 2011; Shukla et al., 2012). WNV is a neurotropic pathogen that is the causative agent of West Nile fever and encephalitis in humans and horses. It is a member of the Japanese encephalitis (JE) virus serocomplex, which includes JEV, Murray Valley encephalitis virus (MVEV) and Saint Louis encephalitis virus (SLEV). WNV is classified within the family *Flaviviridae* and genus *Flavivirus*. WNV is maintained in an enzootic cycle between mosquitoes and birds but can also infect and cause disease in horses and other vertebrate animals (Indenbach, 2001; Knipe and Howley, 2001; Faggioni et al., 2014).

The genome is composed of an 11 kb single open reading frame without a polyadenylation tail (Khromykh et al., 2001; Friebe and Harris, 2010; Colpitts et al., 2012; Mehul et al., 2013). The RNA of WNV is translated into a single polyprotein that is post-translationally cleaved by host and viral proteases into three structural (capsid, envelope, and premembrane) and seven non-structural (NS1, NS2A, NS2B, NS3, NS4A, NS4B, and NS5) proteins. The structural proteins are encoded by the 5′ end of the genome, which are essential for viral entry, fusion and encapsidation of the viral genome during assembly, and envelope protein (53 kDa) is the major protein on the surface of Flavivirus.

WNV infection is diagnosed by serological tests and heamagglutination inhibition test commonly used for demonstration of a 4-fold increase or decrease of antibody titer in serum samples. MAC (IgM-antibody capture) ELISA is routinely used for the acute WNV infection diagnosis in humans (Sambri et al., 2013). The commercially available WNV specific monoclonal antibody (MicroBix Biosystem INC, Canada) used for the detection of the WNV in antibody capture ELISA (Johnson et al., 2000; Martin et al., 2000; Hunt et al., 2002). Plaque reduction neutralization (PRNT) test is very important in confirmation of virus isolates (Martin et al., 2000). Additionally, various molecular based diagnostic methods have been applied for the diagnosis of WNV including traditional RT-PCR (Shi et al., 2001), real-time PCR-based assays, such as TaqMan RT-PCR (Lanciotti and Kerst, 2001; Centers for Disease Control and Prevention, 2002) and nucleic acid sequence-based amplification (NASBA) (Compton, 1991; Kumar et al., 2018). Gold standard method for virus detection is isolation; but it is time consuming and tedious method. Most acceptable method for routine diagnosis of WNV infection is CDC reported TaqMan real-time RT-PCR, which is consider as gold standard method for detection of WNV infection. However, all of these nucleic acid amplification methods have some drawbacks of requiring either a sophisticated instrument for amplification or a complicated method for amplified product detection (Leone et al., 1998). Owing to the problems associated with the current screening systems, it is widely accepted that test results should be confirmed by more than one type of assay. More techniques are therefore needed to complement those already existing techniques.

A number of isothermal gene amplification techniques such as loop-mediated isothermal amplification (LAMP) (Notomi et al., 2000), helicase-dependent amplification (HDA), nucleic-acid-sequence-based amplification (NASBA) (Shi et al., 2001), cross**-**priming amplification (CPA) (Meng et al., 2016), and self-sustained sequence replication reaction (3SR) (Muller et al., 1997) have been reported during past decades (Gupta et al., 2017). These techniques have their own limitations with respect to optimizing the reaction conditions. LAMP assay comprises 6 sets of primers for amplification, HDA require additional enzymes, such as DNA helicase for achieving denaturation of double-stranded DNA and it takes 90 min for completion of the reaction, CPA requires complexity in primer designing and 3SR requires different incubation temperature for completion of the reaction.

In the present study, we have developed the RT-PSR isothermal gene amplification technique for the detection of WNV. The PSR assay was originally described by (Liu et al., 2015) which uses only one set of primers and one enzyme. The PSR method has the advantages of high specificity, sensitivity and rapidity under isothermal conditions over other established gene amplification methods. Until now, the PSR technique has been developed for bacteria (Liu et al., 2015, 2018) yeast (*Candida albicans)* and DNA viruses (canine parvovirus and Bovine herpesvirus-1) (Xiaoqun et al., 2016). PSR approach is based on strand displacement activity of *Bst* DNA polymerase enzyme, isolated from *Bacillus stearothermophillus*.

However, no reports are available on application of the PSR method for detection of RNA viruses. In the present study, we have developed an RT-PSR assay for rapid and real-time detection of WNV where amplification is achieved by incubating with buffer and other components with viral RNA in the presence of *Bst* DNA polymerase at a constant temperature of 63°C for 1 h. Results were interpreted by real-time monitoring in a turbidimeter and visually detected using SYBR Green I dye.

## Materials and Methods

### Cells and Virus Strains

Vero cells (African Green Monkey Kidney epithelial cells) were obtained from National Center for Cell Science (NCCS) Pune, India. The cells were maintained in Eagle Dulbecco's minimum essential medium (Sigma, USA) supplemented with 10% fetal bovine serum (Sigma, USA), Trypsin-EDTA solution (Himedia, India) and antibiotic-antimycotic solution (Conc. 100 mg ml penicillin and 100 mg/ml streptomycin) (Sigma, USA) at 37°C in humified atmosphere with 5% CO_2_ incubator. The WNV (Eg101 strain) used in the present study was obtained from the Institute of Tropical Medicine, Nagasaki, Japan. WNV was propagated by regular passaging in *Aedes albopictus* C6/36 cell lines. The virus was titrated by plaque assay in Vero cells in accordance with the standard protocol (Igarashi, 1978). The viruses utilized in the present study were WNV, JE, SLE, Yellow fever, Dengue, Chikungunya, Ross River, Measles, Mumps, and Rubella.

### Clinical Samples

The serum and plasma samples (*n* = 107) used in this study were collected from the Department of Microbiology, Aravind Eye Hospital, Madurai (TamilNadu) India, from patients with signs of acute posterior uveitis and onset of febrile illness symptoms suspected to have WNV infections during December 2012. The acute phase serum samples collected between days 1 and 9 after the onset of symptoms were used for evaluation. The other clinical symptoms of the patients include fever, ocular manifestation muscle weakness, and poliomyelitis-like flaccid paralysis (Shukla et al., 2012). The patient study was approved by the institutional ethics committee of Aravind Eye Hospital, Madurai, India.

### RNA Extraction

The genomic viral RNA was extracted from 140 μl of WNV infected cell culture supernatant and patient serum and plasma samples using QIAamp Viral RNA Mini Kit (Qiagen, Germany), according to the manufacturer's protocol. The RNA was eluted in 50 μl of elution buffer using QIA spin columns and stored at −80°C until further use.

### Primer Design

WNV specific RT-PSR primers were designed using the DNA STAR software program. Primers were designed using the nucleotide sequence of the env gene of WNV (Accession no. AF260968) with 20–22-bases oligonucleotide sequence was added at the 5′ end to primers. High-performance liquid chromatography (HPLC) grade primers were procured from Chromous Biotech Ltd, Bangalore, India. The details of the oligonucleotide primers used for amplification of the env gene of WNV are given in Table 1.

**Table 1:** Details of RT-PSR Primer set designed for rapid detection of WNV.

**Name of primers**	**Genome position accession no. [AF260968]**	**Primer sequences**
WNV PSR-forward primer	1,268–1,288	5′ acgattcgtacatagaagtatagAGATACAGCTTGGGACTTTG 3′
WNV PSR-reverse primer	1,340–1,359	5′ gatatgaagatacatgcttagcaCAACTGCGAGAAACGTGAG 3′

### *In vitro* Transcription (IVT)

To determine the detection limit of RT-PSR, IVT was carried out. Briefly, full length cloned env gene in PET 28a+ vector was used for IVT. The plasmid was extracted from an overnight grown culture of kanamycin resistant recombinant clone using the plasmid extraction kit (Qiagen, Germany). Plasmid was confirmed using env gene specific primer set by conventional RT-PCR. RT-PCR amplified product was used as the template for IVT. *In-vitro* RNA was synthesized using the Megascript-T7 transcription kit (Invitrogen, USA) according to the manufacturer's protocol. Finally, the RNA pellet was resuspended in nuclease free water. Copy number was determined using the following formula-

$$\begin{array}{l} \text{Y molecules}/\mu \text{l}=\;(\text{X g}/\mu \text{l RNA}/\;[\text{transcript length in nucleotides}\\ \;\times \;340])\text{ x }6.022\text{ x }10^{\text{23}} \end{array}$$

WNV RNA was quantified using a Nanodrop ND-1000 spectrophotometer (Thermo Scientific, Germany). Further 10-fold serial dilutions of the RNA transcript were used for detection of sensitivity and construction of standard curve using the Tp values obtained against the known concentration of serially diluted RNA.

### Reverse Transcription Polymerase Spiral Reaction (RT-PSR Assay)

The RT-PSR assay was carried out at 63°C for 60 min using a real-time turbidimeter instrument (LA-200, Teramecs, Japan). The RT-PSR reaction was performed in 25 μl reaction volume using 2.5 μl 10 × ThermolPol reaction buffer (New England Biolabs, USA) (containing 20 mM Tris-HCl, 10 mM (NH_4_)_2_SO_4_, 2 mM MgSO_4_, 10 mM KCl, 0.1% Tween 20) 0.8 M Betaine (Sigma, USA), 6 mM MgSO_4_ (Sigma, USA), 1.0 μl *Bst* DNA polymerase large fragment (New England Biolabs, USA), 1.4 mM each deoxynucleotide triphosphate (Sigma, USA), 4.0 μM for both forward and reverse primer and 2.5 μl (40 ng) of template. The RT-PSR assay was also optimized the effect of different primer and template concentrations. The effect of higher concentrations of RNA templates in the RT-PSR reaction was also observed by real-time monitoring.

### Reverse-Transcription Polymerase Chain Reaction (RT-PCR)

To compare the clinical sensitivity of the RT-PSR method, RT-PCR was performed using env gene specific reported primers for WNV [Forward: 5′TGGATTTGGTTCTCGAAGG3′ genome position (1,228–1,046) and reverse: 5′GCTCAGCACGTTTGTCATT3′ genome position (1,228–1,210)] (Parida et al., 2004). The amplification was carried out in total reaction volume of 25 μl using a one-step RT-PCR kit (Qiagen, Germany) with 20 nM of forward and reverse primers and 2.5 μl (40 ng) of RNA. The thermal profile of RT-PCR reaction was as follows: RT-step at 50°C for 30 min, denaturation at 94°C for 10 min, followed by 35 cycles of 94°C for 1 min, 55°C for 1 min, 72°C for 1 min and final extension cycle at 72°C for 10 min.

The amplified product was analyzed by agarose gel electrophoresis on a 2% agarose gel (Sigma, USA). The DNA was visualized by ethidium bromide staining and imaged using gel Doc system (Bio-Rad, USA).

### TaqMan Real-Time RT-PCR

For comparative evaluation of the RT-PSR assay, TaqMan real-time RT-PCR was performed using WN-3′NC gene primers WN-3′NC Forward: CAGACCACGCTACGGCG (genome position (10,668–10,684), WN-3′NC reverse: CTAGGGCCGCGTGG (genome position (10,770–10,756) and a WN 3′NC-probe: TCTGCGGAGAGTGCAGTCTGCGAT (genome position 10,691–10,714). The amplification was carried out with total reaction volume of 25 μl using Ag Path-ID one-step RT-PCR reagents (Thermo Fisher, USA) performed in real**-**time instrument (AB Biosystems USA) with 20 nM of forward and reverse primers along with 50 nM probe and 2.5 μl (40 ng) of RNA according to the manufacturer's protocol. The thermal profile of TaqMan real**-**time RT-PCR consists 1 cycle of reverse transcription reaction 50°C for 30 min and 95°C for 10 min and 40 cycles of 95°C for 15 s and 60°C for 1 min.

### Analysis of RT-PSR Product

The RT-PSR amplified products detected by two methods either by real**-**time turbidity monitoring or direct visual detection under UV light using SYBR Green I dye.

### Real-Time Monitoring of RT-PSR

RT-PSR reaction was carried out in the Real-time turbidimeter that measures the optical density through spectrophotometric analysis at 400 nm every 6 s. The turbidimeter determined results in terms of time of positivity (Tp; in minutes). The processing of the sample having Tp values of 60 min or less and threshold value above the >0.1 was considered positive while the threshold values that remained fixed at ≥0.1 were considered negative.

### Naked-Eye Visualization

RT-PSR amplified product was visualized by addition of SYBR Green I dye (Invitrogen, USA). A positive reaction emits bright green fluorescence under ultraviolet (UV) light (302 nm) while negative samples remained orange.

### Statistical Analysis

The clinical sensitivity and specificity of the RT-PSR assay were determined and compared to the conventional RT-PCR. The standard deviation among samples was also calculated. Each experiment repeated three times.

## Results

### Temperature Optimization

**Figure 1:**
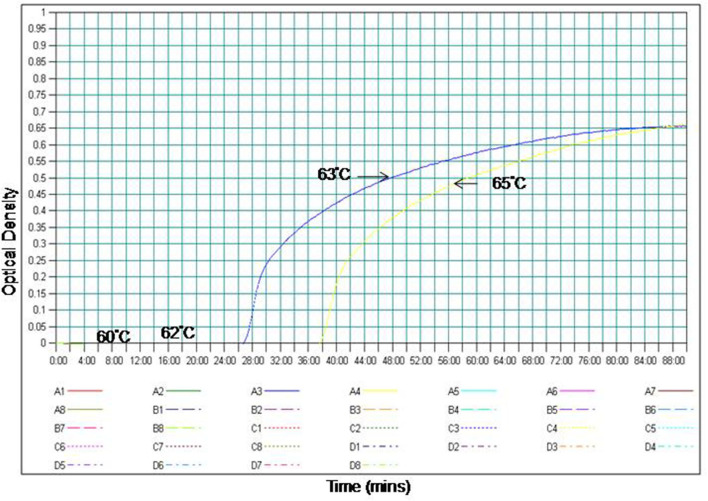
Effect of temperature on the time kinetics of the RT-PSR amplification reaction of WNV Eg101 strain as monitored in real time turbidimeter.

The temperature for RT-PSR assay was optimized using the gradient range from 60 to 65°C (60, 62, 63, 64, and 65°C) for 60 min. At 63°C amplification was observed within 30 min as compared to 65°C where amplification was obtained within 39 min. Therefore, 63°C temperature was selected for the RT-PSR assay to perform further experiments (Figure 1).

### Specificity of RT-PSR

**Figure 2:**
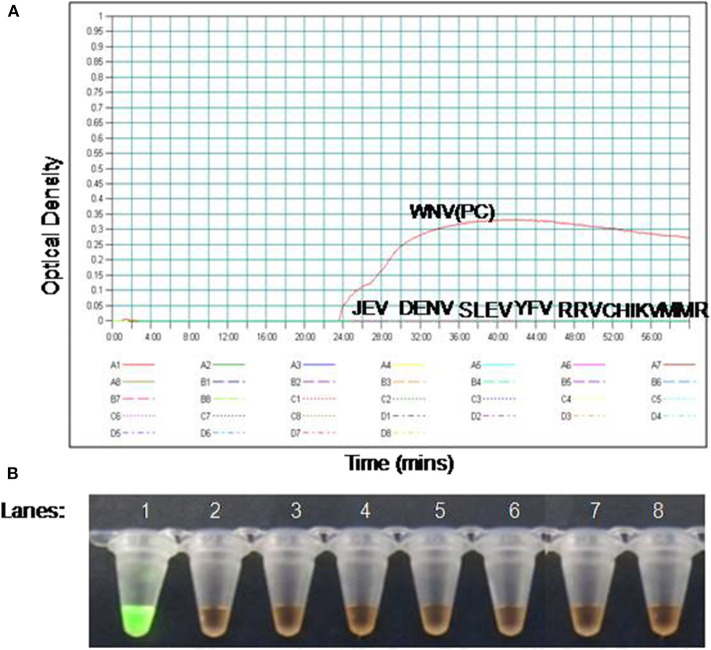
The RT-PSR assay specificity for WNV detection. **(A)** Evaluation by real-time turbidimeter for real time monitoring, **(B)** evaluation by SYBR Green I dye naked eye visualization. Lane 1. West Nile virus (WNV); lane 2. Japanese Encephalitis virus (JEV); lane 3. Dengue virus (DENV); lane 4. Saint Louis encephalitis virus (SLE), lane 5. Yellow Fever virus (YFV), lane 6. Ross River fever virus (RRV); lane 7. Chikungunya virus (CHIKV); lane 8. Measles, Mumps, and Rubella virus (MMR).

The specificity of the PSR assay was established by ruling out the cross**-**reactivity with other viruses. The Eg101 strain of WNV was used as positive control. No cross**-**reactivity was observed with other Flaviviruses, Alphaviruses and Morbillivirus including WNV, JEV, SLEV, YFV, DENV, CHIKV, RRV, and MMR (Figure 2).

### Comparative Sensitivities of RT-PSR and RT-PCR Assay

**Figure 3:**
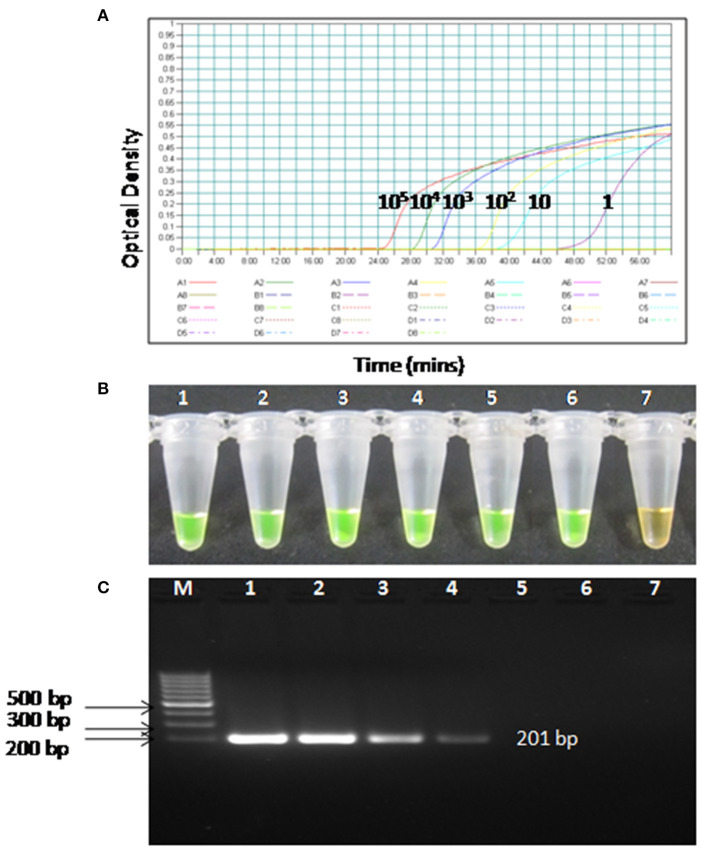
Comparison between sensitivity of RT-PSR and conventional RT-PCR **(A)** real-time kinetics of WNV RT-PSR amplification of the env gene showing the amplification curve with serial 10-fold dilutions of the WNV IVT RNA (10^5^ to 1 copy no.), **(B)** naked eye visualization through SYBR Green I dye, **(C)** RT-PCR performed as the same serial dilution used for RT-PSR and amplified products were stained with ethidium bromide dye. M, 100 bp marker; Lane 1–6, IVT serial dilution (10^5^ to 1 copy no.) Lane 7, Negative (without template) control.

**Figure 4:**
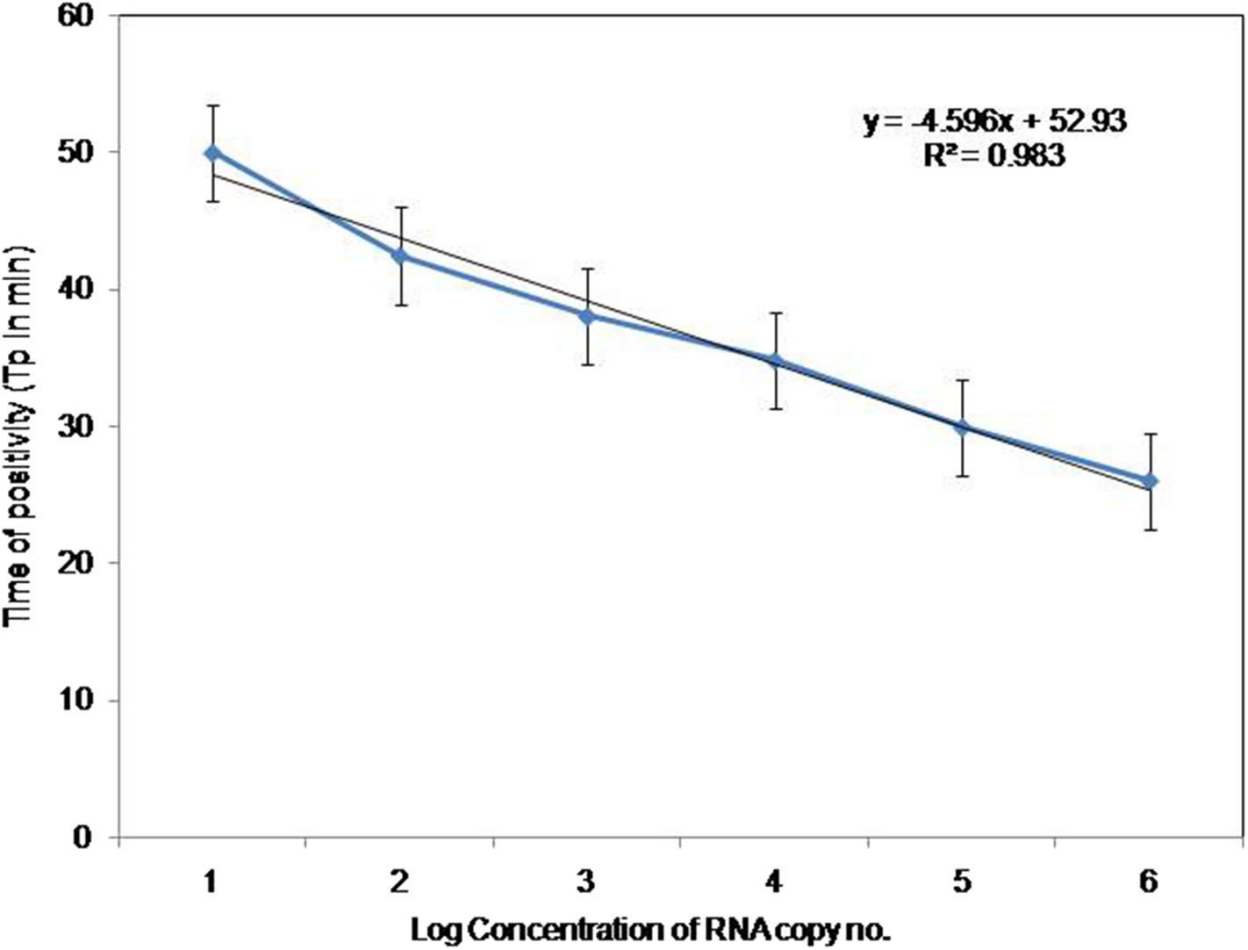
Standard curve for RT-PSR Assay. Env RT-PSR specific standard curve as generated from the amplification plot between different copy number of the template (serial 10-fold dilution from 10^5^ to 1 copy number) and Tp. The Tp value shown here is the average of triplicates run for each concentration.

To compare the sensitivity of the RT-PSR assay with conventional RT-PCR, 10-fold serial dilutions ranging from 10^5^ to 1 copy were tested in triplicates. The detection limit of RT-PSR assay was found to be 1 RNA copy. The comparative sensitivity revealed that RT-PSR is 100 times more sensitive than the conventional RT-PCR, which detected 100 RNA copies (Figure 3). The standard curve generated from 10-fold serial dilutions of IVT WNV RNA showed a linear curve with the coefficient of correlation R^2^ = 0.979 (Figure 4).

### Comparative Evaluation of RT-PSR With Conventional RT-PCR and TaqMan Real-Time RT-PCR Assay

**Figure 5:**
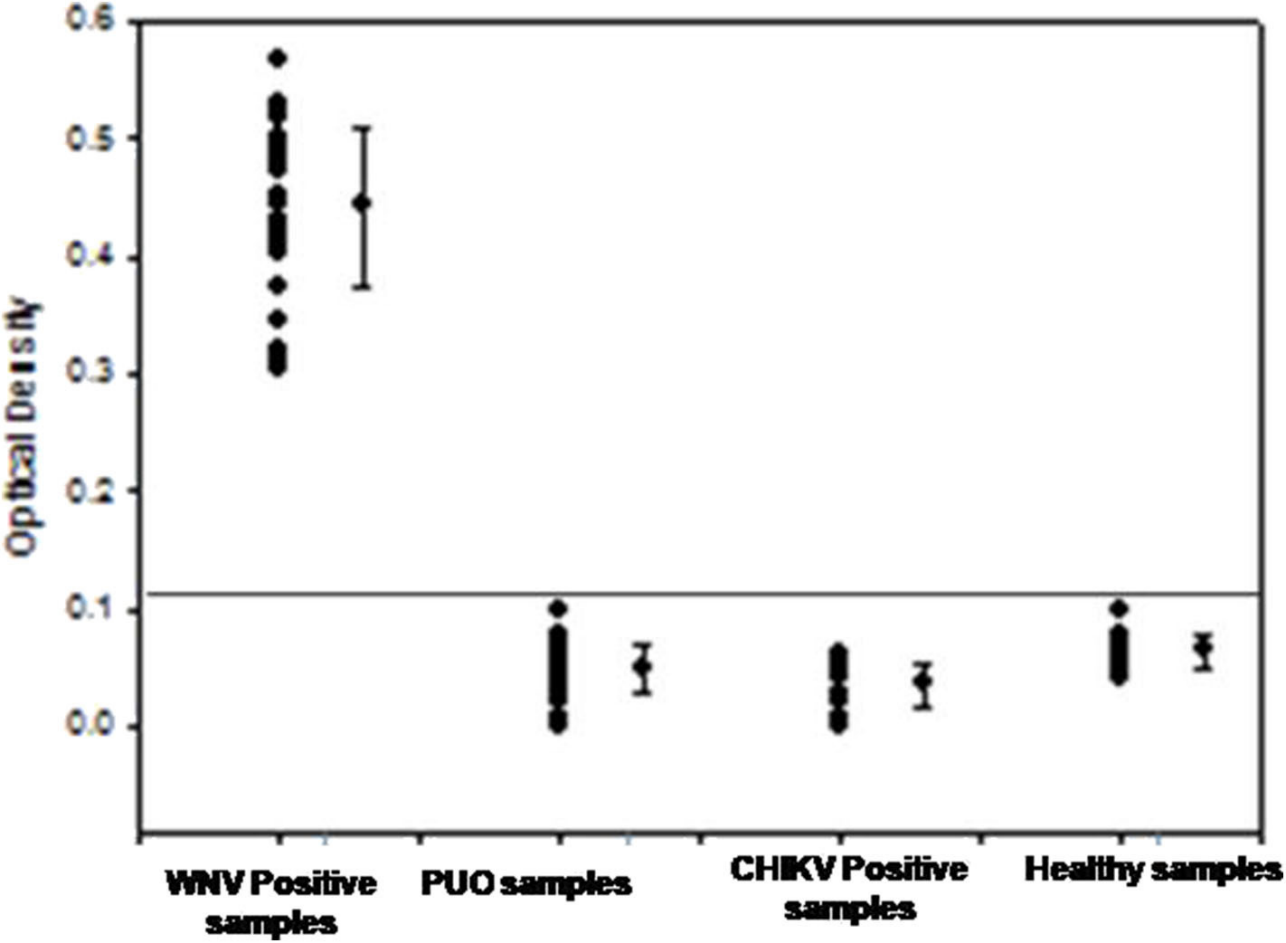
The optical density profile of different patient samples including WNV positive samples, PUO (pyrexia of unknown origin), Chikungunya positive along with samples from healthy volunteers as obtained through the West Nile-specific RT-PSR assay.

To determine the clinical applicability of the RT-PSR method, 107 WNV suspected serum and plasma samples were screened, in addition with a panel of positive serum samples of chikungunya (*n* = 12) and healthy volunteers (*n* = 20), included as negative control (Figure 5).

**Figure 6:**
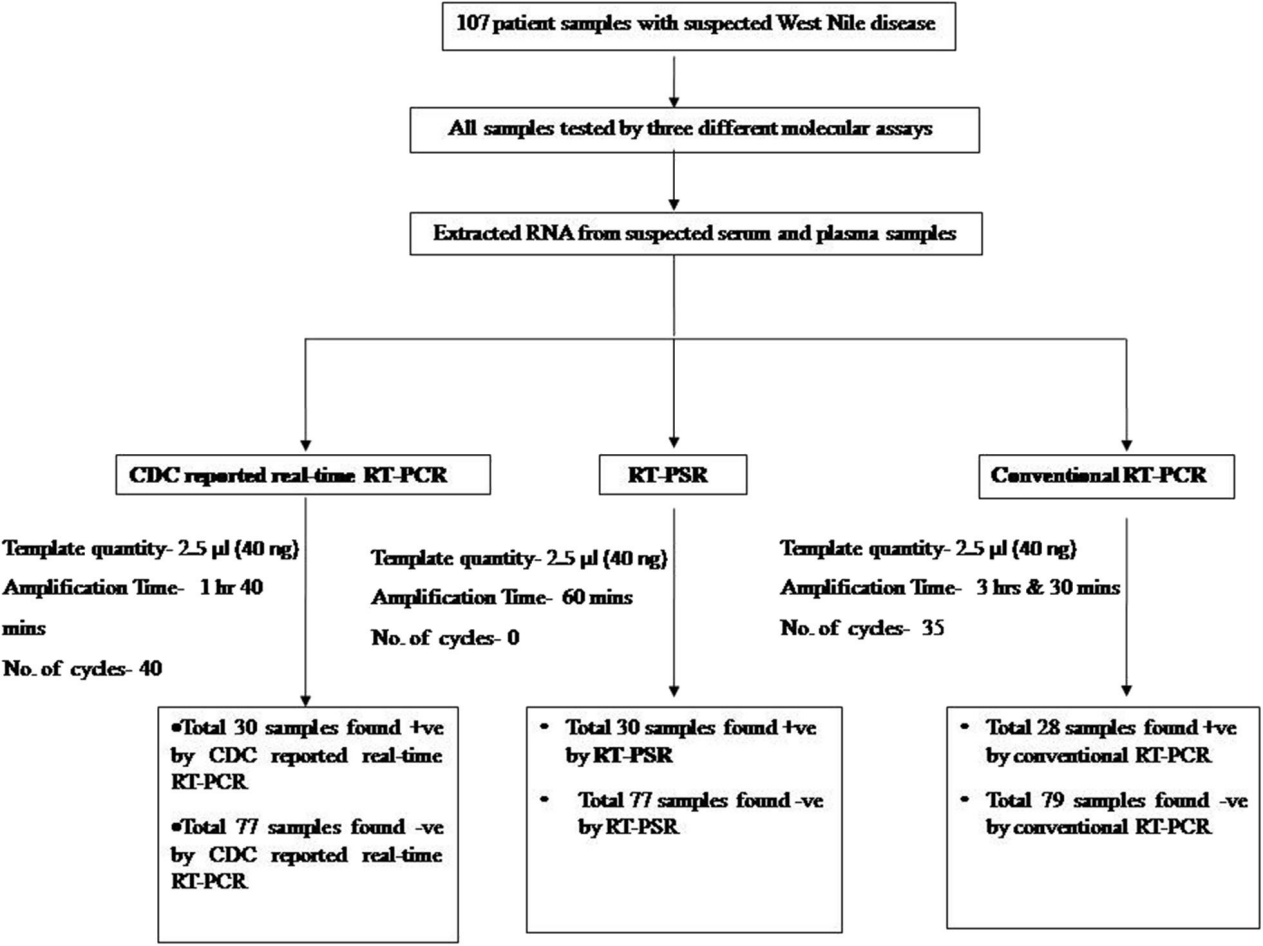
Overview of the WNV-RT-PSR comparative evaluation study.

From the 107 samples, 30 were positive in both RT-PSR and TaqMan real-time RT-PCR, while only 28 were positive by conventional RT-PCR (Table 2). Comparative evaluation between RT-PSR and conventional RT-PCR revealed 98.13% concordance with a sensitivity and specificity of 93.75 and 100%, respectively (Figure 6).

**Table 2:** Comparative evaluation of RT-PSR assay with conventional RT-PCR and real-time RT-PCR assay for the detection of the Env gene of WNV in suspected human-patient serum-plasma samples.

**RT-PSR**	**Conventional RT-PCR**	**Real-time RT-PCR**	**No. of samples**
+	+	+	28
–	–	–	77
+	–	+	2
–	+	–	0

*+, positive; –, negative; Total no. of samples: 107. 28 Samples: Total samples +ve by all three methods. 77 Samples: Total samples –ve by all three methods. 2 Samples: –ve by Conventional RT-PCR methods but +ve by CDC reported Real-Time RT-PCR and RT-PSR methods. 0 Sample: No sample found +ve in Conventional RT-PCR which were –ve by CDC reported Real-Time RT-PCR and RT-PSR methods*.

## Discussion

In India, the presence of antibodies against WNV in humans was first reported in Bombay, in 1952 Banker (1952), and have been reported in other cities more recently (Paramasivan et al., 2003). WNV neutralizing antibodies have been also reported in human serum samples collected from Andhra Pradesh, Orissa, Rajasthan, Madhya Pradesh, Maharashtra, Tamil Nadu, and Karnataka. Similarly, during 1977, 1978, and 1981 WNV seropositive cases were reported from Vellore and Kollar district (Paramasivan et al., 2003). A number of different laboratory based diagnostic methods is used for detection of WNV in clinical samples. Several nucleic acid amplification techniques such as RT-PCR (Higuchi et al., 1993; Shi et al., 2001), TaqMan real-time RT-PCR (Lanciotti and Kerst, 2001) and SYBR Green real-time RT-PCR (Papain et al., 2004) have been reported for rapid detection of WNV. Inspite of the high degree of nucleic acid amplification, these PCR-based methods are expensive and require trained personnel to perform the reaction. In addition, these methods are often complex to adapt for use in clinics with resource limited settings. Therefore, a rapid, sensitive and cost-effective detection method is necessary for proper surveillance of new WNV circulating strains.

TaqMan real-time RT-PCR assay has been reported for detection of WNV from various samples including serum, cerebrospinal fluid (CSF), brain tissue samples from human, field-collected mosquitoes and birds tissue samples (Lanciotti and Kerst, 2001). The real-time PCR based assays have many advantages over conventional RT-PCR methods, presenting rapidity, lower contamination rate, higher sensitivity, quantitative measurement and higher specificity (Heid et al., 1996). These assays are easy to perform, but can be afforded only by referral laboratories with good financial supports. The development of fluorogenic PCR utilizing 5′-3′ nuclease activity of *Taq* DNA polymerase facilitated the removal of post-PCR processing agarose gel electrophoresis (Del et al., 2013). However, all these nucleic acid amplification methods have several drawbacks of requiring either a high precision instrument for amplification or complicated post-PCR processing method for the endpoint detection of amplified products.

These rapid molecular tests might not be the ideal method in basic clinical settings or field based situations. Therefore, it is important to develop simple and rapid molecular tests to overcome the limitations within existing techniques. The present study aimed to develop a RT-PSR assay for a rapid detection of WNV.

The RT-PSR assay is a simple diagnostic tool in which the reaction is performed in a single tube by mixing thermolpol buffer, primers and DNA polymerase followed by incubation at 63°C for 60 min. Since the reaction is performed at a constant temperature, an energy intensive thermal cycler is not needed. Moreover, the positive results could be determined through a visual color change. The sensitivity of RT-PSR was 1 RNA copy when compared to 100 RNA copies in conventional RT-PCR. The assay was evaluated on 107 clinical samples. No cross**-**reactivity was observed with any of the Flaviviruses, Alphaviruses and Morbilliviruses tested. Comparative sensitivity of RT-PSR and RT-PCR revealed 98.13% concordance. PSR has so far been used for detection of a recombinant plasmid containing a blaNDM-1 gene in *E. coli* BL21 bacteria (Liu et al., 2015; Wei et al., 2015, 2018), yeast *Candida albicans* (Xiaoqun et al., 2016), canine parvovirus and Bovine Herpesvirus 1 (Gupta et al., 2017; Javed et al., 2018). One important characteristic of this isothermal gene amplification technique is the field based application, by using SYBR Green I dye-mediated naked eye visualization.

This is low cost, rapid, simple, and sensitive technique, since gene amplification can be performed in a heating block/water bath, and visualization of the green fluorescence light can be performed by using a simple UV hand-held torch (Parida et al., 2011). Currently, the cost of a RT-PSR reaction is estimated to be 47.46 Rupees per sample test. The light green fluorescence can be observed by using a simple UV hand-held torch. The test is rapid and amplification can be achieved within 60 min as compared to the 3–4 h required by conventional gene amplification techniques. The method is sensitive, specific and enables to detect low copy number of virus, mostly in some cases that would be missed by conventional RT-PCR techniques. These findings suggest that the env gene-specific RT-PSR assay is an important diagnostic tool for rapid and real-time detection of WNV.

## Conclusion

RT-PSR is a rapid, sensitive and specific method to detect the RNA virus WNV in clinical samples with comparison to the RT-PCR method. The method is simple, low cost and rapid, performed under isothermal conditions over 1 h and can be both quanitified through turbidity or though UV color change, making it an attractive option for use in the field.

RT-PSR has a short performance time, which is a desirable characteristic amenable for samples testing in limited infrastructure settings in endemic rural areas during an outbreak. We believe it will enable early detection of the WNV infection and help controlling its spread among humans.

## Data Availability Statement

The datasets generated for this study can be found in NCBI, accession numbers JN591727 to JN591753.

## Ethics Statement

The studies involving human participants were reviewed and approved by Aravind Eye Hospital, Madurai, India. The patients/participants provided their written informed consent to participate in this study.

## Author Contributions

JK: conceptualization, data analysis, review, and supervision of MS. PT: standardization, evaluation of the assay, data analysis, and manuscript writing. SP: review and editing of MS writing. SS: review of MS. All authors: read and approved the final manuscript.

## Conflict of Interest

The authors declare that the research was conducted in the absence of any commercial or financial relationships that could be construed as a potential conflict of interest.
